# From Gardner fibroma diagnosis to constitutional *APC* mutation detection: a one‐way street

**DOI:** 10.1002/ccr3.1065

**Published:** 2017-08-10

**Authors:** Claudia Santoro, Teresa Giugliano, Delfina Bifano, Carolina D'Anna, Vittoria D'Onofrio, Silverio Perrotta

**Affiliations:** ^1^ Dipartimento della Donna del Bambino e della Chirurgia generale e specialistica Università degli Studi della Campania Luigi Vanvitelli Naples Italy; ^2^ Dipartimento di Biochimica Biofisica e Patologia Generale Università degli Studi della Campania Luigi Vanvitelli Naples Italy; ^3^ Telethon Institute of Genetics and Medicine Pozzuoli Italy; ^4^ Department of Anatomopathology Santobono‐Pausilipon Children's Hospital Naples Italy; ^5^ Department of Pediatric Emergency Santobono‐Pausilipon Children's Hospital Naples Italy

**Keywords:** *APC*, familial adenomatous polyposis, Gardner fibroma

## Abstract

We report a young child without a family history of FAP, who promptly underwent *APC* testing after the histological confirmation of a paraspinal GAF that was not isolated. Our case report reinforces the suggestion advanced by previous authors for an *APC* analysis in all patients with GAF.

## Introduction

The Gardner fibroma (GAF) is a benign soft tissue lesion that most commonly locates in the back and paraspinal region 1. This soft ill‐defined, white rubbery mass is poorly circumscribed and classically contains flecks of yellow entrapped adipose tissue. Males and females are equally affected, with a predilection for the first two decades of life. Immunohistochemistry evaluation of GAF reveals diffuse reactivity for CD34 and nuclear beta‐catenin 2. The beta‐catenin overexpression in GAF may result from activating somatic variants of the *CTNNB1* gene or from biallelic inactivation of the *APC* gene, but the pathogenetic mechanisms are still unknown. Intriguingly, germline mutations of the *CTNNB1* gene cause familiar desmoids syndrome 3, where GAFs could also occur. Mutations in the *APC* gene cause some forms of familiar desmoids syndrome 4 and GS as well as FAP.

## Case Report

An 18‐month‐old female who had recently undergone resection of a GAF was referred to us for genetic counseling.

She was the only child of nonconsanguineous healthy parents. The patient's father had myelinated retinal nerve fiber persistence. A paternal uncle had multiple myeloma. The paternal grandfather died from Hodgkin lymphoma at 47 years of age, whereas the maternal grandmother was diagnosed to have colonic diverticular disease and one hyperplastic intestinal polyps at 60 years of age. A paternal aunt of the patient's mother also presented a colon cancer at 60 years.

Our patient was born after 39 weeks of gestation. From birth, asymmetric frontal and occipital bossing had been present. At 9 months of age a paraspinal, nontender, slightly mobile, and nodular lesion was noted. An ultrasound revealed that it was a 3 × 0.5‐cm fusiform mass, located in the subcutaneous adipose tissue, without any deep infiltration and vascularization. An additional subcentimetric lesion was touchable in the subcutis of the posterior neck.

**Figure 1:**
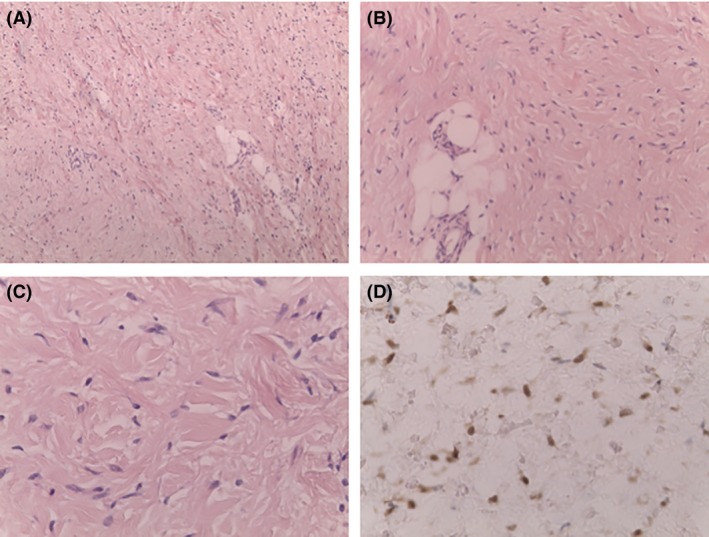
Histological and immunohistochemical findings. (A and B) histologically, the lesion displays hypocellular sheets of haphazardly arranged collagen bands with few interspersed spindle cells; an infiltrative pattern was present at the periphery with entrapment of fat. (C) bland spindle cells were present between collagen fibers separated by clear cracks. (D) beta‐catenin immunohistochemical reactivity is present with a nuclear pattern.

A surgical excision of the paraspinal lesion was performed. The lesion was identified as a poorly circumscribed, rubbery white mass. Histologically, the lesion displayed hypocellular sheets of haphazardly arranged collagen bands with a few interspersed spindle cells (Fig. 1A–B), clear cracks between collagen fibers and rare capillary‐sized vessels (Fig. 1C). An infiltrative pattern was present at the periphery and an entrapment of fat and nerve fibers. At immunohistochemistry, the spindle cells were positive for vimentin, CD34, and nuclear beta‐catenin but negative for muscle specific actin, smooth muscle actin, and desmin (Fig. 1D). These observations were compatible with the diagnosis of GAF.

MRI and ultrasound examinations of the neck identified the small lesion as an additional fibroma.

At clinical examination, at the age of 18 months, the child showed a normal development in terms of growth parameters and cranium circumference. A subtle nodular lesion below the surgical scar was still noticeable. A subcutaneous elliptical lesion involving the right frontal bone, slightly mobile, was also observed. The remainder of the physical examination did not reveal any other skin lesions or clinically evident bone abnormalities. Dental and oral examinations were also normal. Finally, the retinal examination of the child showed no signs of congenital hypertrophy of the retinal pigment epithelium (CHRPE).

Given that GAF had recently been identified as a potential sentinel of familial adenomatous polyposis (FAP) 5, the molecular analysis of the *APC* gene on constitutional DNA was performed in our patient by Sanger sequencing and by multiple ligation‐dependent probe amplification (SALSA MLPA kit P043 APC, lot. C1‐1212). The analysis identified the heterozygous variant c.4391_4394del, p.(Glu1464Valfs*8) of the *APC* gene (NM_000038.4) which is predicted to be deleterious. The mutation was subsequently excluded in her parents. Paternity testing was performed according to the ISO 17025 standard 6.

No somatic *APC* cytogenetic alterations or point mutations in the tumor DNA were investigated.

The recurrence risk for our patient's parents was considered, even if low, to be greater than that of the general population because of the possibility of germinal mosaicism. Recent studies indicated the presence of mosaicism in approximately 15% of parents of apparently sporadic cases 7, 8.

## Discussion

GAFs are rare and well‐defined soft tissue tumors at immunohistochemistry. They tend to occur in childhood and early adulthood 1.

In the last few years, the potential sentinel role of these lesions in FAP has emerged 9. In fact, much attention has been on the extracolonic manifestations of the disease that have a tendency to precede colonic lesions. Other extracolonic lesions were found among these diseases include desmoid‐type and nuchal‐type fibromas, cutaneous epidermoid cysts, osteomas, and Gardner‐associated fibromas 10, 11.

Given that in FAP the onset of intestinal manifestations begins to develop at the age of 16 years 12, a proper and precocious diagnosis and follow‐up is desirable. In the absence of a positive family history, as in our case, FAP diagnosis is made only when polyposis or other more serious FAP complications are present. In particular, it is estimated that about 15–20% of constitutional *APC* variants occur de novo. Especially in these cases, the presence of a clinical sentinel, such as GAF, might help geneticists in making the diagnosis of FAP. A proper histological diagnosis of GAF is clearly of great value in this context.

Wehrli et al. 10 reported the results of an *APC* analysis in 11 children with GAF. Only one of them presented with a GAF in the absence of a positive family history of FAP. The *APC* analysis in this child was negative. In all the other patients, patients who presented a GAF plus a positive family history of FAP and constitutional *APC* mutations were identified. Later, Vieira et al., 5 focused on the role of GAF as a sentinel of FAP. The authors described two infants with both GAF and a previously unrecognized familial history of FAP. Both patients had a biallelic inactivation of the *APC* gene with the constitutional variants c.4687dup, p.(Leu1563Profs*4) and c.5826_5829del, p.(Asp1942Glufs*27), respectively. After genetic counseling, additional family members with FAP features carrying these mutations were identified. In 2016, Dahl et al. 13 instead reported the *APC* molecular analysis results directly performed in a cohort of seven children because of the presence of GAF. None of them had a family history of FAP. Three patients had a heterozygous constitutional mutation in the *APC* gene, and all of them presented with multifocal, large, or surgically unresectable GAF. Four of the seven patients, negative at the *APC* test, had single resectable lesions.

For the patient described here, a genetic diagnosis of FAP was made through the molecular analysis of the *APC* gene from peripheral blood DNA lymphocytes performed on the sole presence of GAF, without a positive family history of FAP. The patient had an additional neck fibroma, which, nevertheless, was not biopsied.

The analysis of her constitutional DNA identified the deleterious variation c.4391_4394del, p.(Glu1464Valfs*8) in the *APC* gene. This is a frameshift mutation predicted to cause a premature stop codon with a strong functional effect on the APC protein. The mutation occurs in the exon 18 of the gene and has already been reported 14, 15.

In a recent study, some authors reviewed all the published literature about the diagnosis of FAP, desmoid tumors, and *APC* mutations. Their data emphasized that mutations in the 5′ (543–713) and 3′ (1310–2011) regions of the *APC* gene are associated with an increased risk of desmoid tumors 16.

In our case the *APC* mutation occurred in the codon 1464.

## Conclusions

Our results, together with those from the recent literature, confirm that GAF really represents a powerful sentinel element for FAP, especially in those cases with multifocal, large, or unresectable lesions 13.

Therefore, genetic counseling is recommended in the presence of GAF, even if isolated, and in the absence of any family history of FAP. It may be advisable to look for constitutional mutations of *APC* in all children presenting with GAF as a first step.

## Consent

Written informed consent was obtained from the parents/patient for publishing the case report for study enrollment and genetic analysis.

## Authorship

CS: followed the patient performing the genetic counseling, coordinated the project, and wrote the manuscript. TG: wrote the manuscript and reviewed the current literature in the matter. BD and DOV: performed the histological diagnosis, provided images and created figures, and revised the manuscript. DAC: wrote the manuscript. PS: critically reviewed the manuscript and coordinated the project.

## Conflict of interest

Nothing to declare.
